# Charing Cross Hospital: Abstract of Clinical Lecture of the Treatment of Empyema

**Published:** 1897-09-04

**Authors:** J. H. Morgan

**Affiliations:** Surgeon to the Hospital


### CHARING CROSS HOSPITAL.

### Abstract of Clinical Lecture on the Treatment of Empyema.

By J. H. Morgan, M.A.Oxon., F.R.C.S., Surgeon to the Hospital.

The case which formed the subject of this lecture was that of a man, aged 35, who was admitted on the 8th inst., with symptoms of a collection of fluid in the right side of the chest.

His clinical history showed that he had had inflammation of the lungs ten years ago, and, though recovering perfectly from this attack, he had had several slight attacks of influenza. The last occurred ten weeks ago, and was followed by pneumonia of the right lung. The lecturer alluded to the fact that these cases of influenza, followed by pneumonia, and giving rise to empyema, seemed to occur in cycles, and during the epidemic of 1893 he had in the course of five months operated upon eight cases in this hospital, all of which recovered. The disease occurred at all ages, and was frequent in children. The most common causes were chills from exposure, pneumonia following influenza, tuberculosis, pyæmia, or septicæmia, often bilateral, abscess extending from below the diaphragm, rupture of hydatids into the pleura, and injuries such as contusions with or without fracture, penetrating wounds from gunshot injuries, &c.

The varieties were two; either the collection was bounded by adhesions of the pleural surfaces, and thus localised, which ensued generally as a result of inflammation of a limited area of lung tissue, or it was diffused over the whole pleural cavity.

The symptoms were: Firstly, those indicating effusion of fluid into the pleura, *e.g.*, dulness, absence of breath sounds and vocal fremitus, bulging of the intercostal spaces, dyspnœa, displacement of viscera, especially the heart, more pronounced when the effusion was on the left side, and immobility of the side affected; secondly, those indicating the formation of pus, rigors, fever, with high evening temperature. These symptoms together indicate the presence of pus in the cavity of the pleura, and all were present in the patient under discussion. He was thin, and had wasted considerably in the last few days; the skin was grey and moist, the breathing rapid and shallow, the breath sounds exaggerated over the left thorax, but absent over the whole of the right. The apex of the heart was not displaced, but the action was rapid and feeble. Five days previous to his admission, the doctor under whose care he had been had withdrawn more than 40 oz. of pus by means of the aspirator, but the chest had rapidly filled again, and a small aspirator had withdrawn some pus on the day of his admission.

The means by which Nature occasionally effects relief in these cases were pointed out, viz., by discharge through the bronchi, or by opening into the stomach or intestines, or by the abscess pointing in the abdominal wall or even in the groin, or presenting on the surface of the chest, generally in front, in the third or fifth interspace. Such solutions were seldom satisfactory, and the results of operative treatment were so encouraging that when once the presence of pus was determined, one of three operations was resorted to: (1) Aspiration. This in the case of small collections, and especially in children, was sometimes sufficient after one or more applications to effect a cure. (2) Free incision. This meant a free opening through skin and muscles in one of the intercostal spaces. The space selected was generally the eighth or ninth, or some other spot overlying any localised collection. Objections to this course were discussed and preference given to the third, viz., (3) removal of a portion of rib. In the case of the patient under discussion an incision had been made down to the seventh rib, and after incising and scraping off the periosteum a piece of about $2\frac{1}{2}$ inches in length had been removed with cutting forceps. By this proceeding a free exit was given to a large quantity of pus, amounting to nearly four pints, and the finger was enabled to explore the condition of the thorax on that side. The lung was collapsed and pushed against the spine and the diaphragm was depressed below its normal level. In the present instance there was less than the usual amount of flocculent lymph, but in order to remove as much as possible some warm water was injected into the cavity, and was then made to escape by turning the patient over upon the affected side. By this means the cavity was rendered as clean as possible, and a drainage tube of the usual form was inserted and the ends of the wound united. This was the usual method of dealing with these large collections both in adults and children.

As was demonstrated by a visit to the patient, the lung had already expanded to a great extent, and it would, it was hoped, regain its normal size and relations, so as to fill up the cavity of the thorax. Should it not do so much might be hoped for by the rising of the diaphragm, and something from the collapse of the thoracic walls.

